# Differential distribution and prognostic value of CD4^+^ T cell subsets before and after radioactive iodine therapy in differentiated thyroid cancer with varied curative outcomes

**DOI:** 10.3389/fimmu.2022.966550

**Published:** 2022-08-26

**Authors:** Zhi-Yong Shi, Sheng-Xiao Zhang, Cai-Hong Li, Di Fan, Yan Xue, Zhe-Hao Cheng, Li-Xiang Wu, Ke-Yi Lu, Zhi-Fang Wu, Xiao-Feng Li, Hai-Yan Liu, Si-Jin Li

**Affiliations:** ^1^ Department of Nuclear Medicine, First Hospital of Shanxi Medical University, Taiyuan, China; ^2^ Collaborative Innovation Center for Molecular Imaging of Precision Medicine, First Hospital of Shanxi Medical University, Taiyuan, China; ^3^ Department of Rheumatology, Second Hospital of Shanxi Medical University, Taiyuan, China; ^4^ Key laboratory of Cellular Physiology, Ministry of Education, Shanxi Medical University, Taiyuan, China

**Keywords:** differentiated thyroid cancer, efficacy assessment, CD4^+^ T cell subsets, radioactive iodine therapy, prognosis

## Abstract

Differentiated thyroid cancer is the most frequently diagnosed endocrine tumor. While differentiated thyroid cancers often respond to initial treatment, little is known about the differences in circulating immune cells amongst patients who respond differently. A prospective study of 39 patients with differentiated thyroid cancer was conducted. Serum thyroglobulin levels and thyroid and immunological functions were tested before and after radioactive iodine treatment (RAIT). Efficacy assessments were performed 6 to 12 months after radioactive iodine treatment. Most patients showed an excellent response to radioactive iodine treatment. Before radioactive iodine treatment, the excellent response group had considerably fewer circulating CD4^+^ T cell subsets than the non-excellent response group. Both the excellent response and non-excellent response groups had considerably lower circulating CD4^+^ T lymphocyte subsets 30 days after radioactive iodine treatment, but those of the excellent response group were still lower than those of the non-excellent response group. All circulating CD4^+^ T cell subsets in the excellent response group rose by varying degrees by the 90th day, but only Treg cell amounts increased in the non-excellent response group. Interestingly, in the non-excellent response group, we noticed a steady drop in Th1 cells. However, the bulk of circulating CD4^+^ T cell subsets between the two groups did not differ appreciably by the 90th day. Finally, we discovered that CD4^+^ T cell subsets had strong predictive potential, and we thus developed high-predictive-performance models that deliver more dependable prognostic information. In conclusion, in individuals with differentiated thyroid cancer, there is great variation in circulating immune cells, resulting in distinct treatment outcomes. Low absolute CD4^+^ T cell counts is linked to improved clinical outcomes as well as stronger adaptive and resilience capacities.

## 1 Introduction

Thyroid cancer is the most frequently diagnosed endocrine cancer, with an annual incidence of 8–9 cases per 100,000 persons worldwide. Differentiated thyroid cancer (DTC) accounts for more than 90% of all thyroid cancers (1). DTC has become more widespread in the last 10 years as imaging technology has improved, and people’s lifestyles have changed (2–4).

The most commonly utilized first-line treatment option for individuals with DTC is radioactive iodine therapy (RAIT) following complete thyroidectomy (5). In addition, the American Thyroid Association recommends that DTC patients, particularly those at intermediate and high risk, undergo long-term active follow-up after RAIT (6). At 6–12 months, the initial therapeutic response should be evaluated. Serum thyroglobulin (Tg) levels in the blood and lymph node invasion are two major predictors of tumor development that must be monitored during long-term follow-up of DTC patients who have received RAIT (7–9). Although this management strategy has proven effective, some patients continue to face tumor recurrence and metastasis, which substantially influences survival (2, 10–14). Previous research has suggested that thyroglobulin (Tg) or preoperative anti-thyroid peroxidase antibody (TPOAb) levels are prognostic indicators for DTC recurrence (7, 15). However, their predictive power is limited. This necessitates deeper investigations into novel predictive tools.

Cancer immune surveillance mechanisms can eliminate aberrant cells from the body, preventing tumor formation. Tumor incidence and development in the body result from a series of dynamic and complicated interactions between the immune system and tumor cells, including clearance, balance, and escape (16–18). Tumors are the result of immunological escape. In addition, tumor cells that developed from normal cells appear to have a higher chance of developing immune evasion mechanisms, including tumor cell self-modification, alterations in metabolism, and changes in the tumor microenvironment. At a certain point, the body’s anti-tumor immune system will fail in preventing further development, and tumor cells will grow and divide uncontrollably (19). Thus, it is important to increase T cell recognition of tumor cells for anti-tumor immunity.

CD8^+^ cytotoxic T cells, CD4^+^ T helper cells, and dendritic cells are important biological mediators of cancer immune surveillance. CD4^+^ T cells primarily consist of helper T (Th) cells, which adhere to the non-polypeptide portion of major histocompatibility complex class II molecules and participate in signal transduction using T cell antigen receptors to identify antigens, thereby activating CD8^+^ T cells (20–22). CD8^+^ T cells can release IFN-γ to destroy tumor cells directly (23). These findings indicate that CD4^+^ T cells are important in anticancer immunity (24–26). Currently, tumor immunotherapies seek to activate the body’s immune system to produce a potent anti-tumor immune response. However, current methods only show good outcomes in a limited number of patients (27–29). This might be due to a lack of understanding of the distribution and function of CD4^+^ T cell subsets (Th1, Th2, Th17, and regulatory T cells (Treg cells)) in the tumor microenvironment and circulatory system (30, 31).

Therefore, the purpose of this study was to examine the differences in the dynamic distribution of circulating CD4^+^ T cell subsets before and after RAIT in DTC patients who had different therapeutic outcomes, as well as to assess the diagnostic and predictive potential of CD4^+^ T cell subsets (before and after RAIT) relative to serum Tg before RAIT. Furthermore, we designed and evaluated multiple predictive models to improve the prognosis of DTC patients.

## 2 Materials and methods

### 2.1 Patients

Between January 2021 and August 2021, 79 participants consisting of 39 DTC patients and 40 age- and sex-matched healthy controls (HC) were recruited from Shanxi Medical University’s First Hospital. According to the EANM guide, all DTC patients were treated with thyroidectomy and radioactive iodine therapy (RAIT). Patients with acute inflammatory or autoimmune disorders, chronic inflammation or other malignancies, or other conditions that might cause altered immunity were excluded from this study. In the three months before recruitment, none of the patients had received glucocorticoids or immunosuppressive medicines. All patients had peripheral blood samples drawn, and thyroid and immune function tests were performed on day 0 before RAIT, as well as on 30 and 90 days after RAIT. Free T3 (FT3), free T4 (FT4), thyroid-stimulating hormone (TSH), and thyroglobulin (Tg) levels were all assessed using thyroid function assays (SN-697, Shanghai Nuclear Research Institute Rihuan Photoelectric Instrument Co., Ltd, China). Immune function testing included evaluating CD4^+^ T cell subset levels, using the detection methods described in the following section (**Figure 2A**). Venous blood was taken prior to RAIT for standard analyses of lymphocytes, neutrophils, four blood lipids (triglyceride (TG), total cholesterol (TC), high density lipoprotein cholesterol (HDL-C), and low density lipoprotein cholesterol (LDL-C)), and liver function. The institutional review board of Shanxi Medical University’s First Hospital in China approved this study.

### 2.2 Flow cytometry

Flow cytometry was used to measure the total number of lymphocytes in each of the subpopulations (CD3^+^ T/CD4^+^ T/CD8^+^ T/B/NK cells) which were isolated from peripheral blood samples. Blood cells were collected by flow cytometry using a BD FACSCalibur platform (BD Biosciences, Franklin Lakes, NJ, USA) and quantified using MultiSET software (BD Biosciences) after 50 µL of EDTA-anticoagulated venous blood was placed into A and B Trucount tubes (BD Biosciences). The CD3^+^/CD4^+^/CD8^+^ T cell subsets were identified using the conjugated monoclonal antibodies fluorescein isothiocyanate (FITC)-CD3, allophycocyanin (APC)-CD4, peridinin chlorophyll protein (PerpCP)-CD45, and phycoerythrin (PE)-CD8 (20210301; Beijing Tongsheng Shidai Biotech Co., Ltd, Beijing, China). For B and NK cell populations, the monoclonal antibodies FITC-CD3, APC-CD19, PerpCP-CD45, and PE-CD16+CD56 were used (20210726; Beijing Tongsheng Shidai Biotech Co., Ltd.). CD4^+^ T cell subsets (Th1/Th2/Th17/Treg cells) were then detected and Th1/Th2/Th17 cells were stimulated with PMA and ionomycin. These cells were then labeled with FITC-conjugated anti-CD4 antibodies (20210604; Beijing Tongsheng Shidai Biotech Co., Ltd.), fixed, permeabilized using 1 mL freshly prepared fixation/permeabilization buffer, and then stained with APC-conjugated anti-IFN-γ (C7048040521503; Beijing Tongsheng Shidai Biotech Co., Ltd.)/PE-conjugated IL-4 (C7048040521503; Beijing Tongsheng Shidai Biotech Co., Ltd.)/PE-conjugated IL-17A antibodies (0314403; Beijing Tongsheng Shidai Biotech Co., Ltd.). Cell surfaces were stained with FITC-conjugated anti-CD4 and APC-conjugated anti-CD25 (0139110; Beijing Tongsheng Shidai Biotech Co., Ltd.), before being fixed and permeabilized in a 4°C incubator with 1 ml Cytofix/Cytoperm reagent. This was followed by staining with anti-Foxp3-PE (2429732; Beijing Tongsheng Shidai Biotech Co., Ltd.) for 30 min, according to the manufacturer’s protocol for Treg cells. CellQuest software version 6 was used to compute the proportion of each subpopulation, and the absolute number of cells in each subpopulation was estimated as follows: absolute cell number = percentage of positive cells in each subgroup × absolute counts of CD4^+^ T cells (cells/μL).

### 2.3 Response assessment

All patients were assessed for treatment effectiveness within 6–12 months of receiving RAIT. According to the 2015 American Thyroid Association guidelines, patients were classified as: (I) excellent response (ER) (i.e., inhibitory Tg < 0.2 µg/L or stimulatory Tg < 1 g/L, negative imaging examination); (II) indeterminate response (IDR) (i.e., 0.2 g/L ≤ inhibitory Tg < 1 g/L or 1 g/L ≤ stimulatory Tg < 10 g/L, negative imaging examination); (III) biochemical incomplete response (BIR) (i.e., 1 g/L < inhibitory Tg or 10 g/L < stimulatory Tg, negative imaging examination); or (IV) structural incomplete response (SIR) (i.e., structural disease testing independent of Tg and TgAb results).

All individuals in this study were separated into two groups: efficacy response (ER) or non- efficacy response (NER), which included IDR, BIR, and SIR classified patients.

### 2.4 Statistical analysis

All statistical analyses were carried out using SPSS 22.0 (SPSS Inc., Chicago, IL, USA) and/or GraphPad Prism 9 software (GraphPad Software, San Diego, CA, USA). Simple and relative frequencies were used for analysis of categorical variables. Median, average, standard deviation, and range values were used for analysis of continuous variables. Continuous variable data were tested for normality using the normality test. Student’s *t*-tests and the Mann–Whitney U tests were used for normally distributed continuous data and non-normally distributed continuous variables, respectively. Bonferroni correction was used to correct for multiple variables. One-way ANOVA was used for normally distributed continuous variables when comparing three or more populations, and the Kruskal-Wallis rank-sum test was used for non-normally distributed continuous variables. Repeated measures ANOVA tests were employed for factors that change over time. To compare pre- and post-treatment results, paired *t*-tests were performed. The predictive efficacy of Tg and CD4^+^ T cell subsets was assessed using univariate logistic regression analysis. Predictive ability was assessed using multiple regression analysis. To distinguish ER from NER, receiver operating characteristic (ROC) curves were drawn, and the optimal cut-off value for each index was selected. The area under the curve (AUC) was determined using a 95% confidence interval (CI). Statistical significance was set at *p* < 0.05.

## 3 Results

### 3.1 Comparison of clinical data

The clinical data of the two groups are summarized in **Table 1**. The ER group consisted of 21 patients with an average age of 43.95 ± 12.54 years, 71.4% (*n* = 15) of whom were female. The NER group included 18 patients with an average age of 40.77 ± 2.24 years, with 61.12% (*n* = 11) being female. There was no significant difference between the two groups in terms of age, gender, tumor volume, number of lesions, BMI, or partial testing (*p* > 0.05; **Table 1**). Furthermore, there were no statistically significant changes between the two cohorts in the four blood lipids (TC, TG, HDL-C, and LDL-C), lymphocytes, neutrophils, ALT, or AST (*p* > 0.05; **Figure S1**). Several studies have shown that these measures are strongly associated with thyroid cancer (32–34); however, we did not find significant differences between the two cohorts.

**Table 1:** Comparison of general clinical data of the two groups of patients.

	ER (n=21)	NER (n=18)	P
**Age (year), median (range)**	46 (20-77)	37.5 (24-59)	0.386
**Tumor size (cm), mean ± SD**	1.35 ± 0.89	1.31 ± 1.02	0.908
**Gender, n (%)**			0.734
Male	6 (28.57)	7 (38.88)	
Female	15 (71.43)	11 (61.12)	
**Number of lesions, n (%)**			0.748
Single lesion	10 (47.61)	7 (38.88)	
Multiple lesions	11 (52.39)	11 (61.12)	
**BMI**	20.19 ± 3.11	21.01 ± 4.29	0.497
**Leukocyte (10’9/L), mean ± SD**	5.30 ± 1.16	5.81 ± 1.39	0.224
**Red blood cells (10’12/L), mean ± SD**	4.80 ± 0.55	5.01 ± 0.42	0.178
**Hemoglobin (g/L), mean ± SD**	141.09 ± 21.24	146.8 ± 19.69	0.39
**Urea (mmol/L), mean ± SD**	3.72 ± 1.05	4.04 ± 1.29	0.392
**Cr (μmol/L), mean ± SD**	70.94 ± 11.14	71.17 ± 17.98	0.962
**UA (μmol/L), mean ± SD**	309.00 ± 86.14	364.00 ± 112.97	0.093

BMI, Body Mass Index; Cr, creatinine; UA, uric acid; ER, excellent response; NER, non-excellent response. These blood parameters are reference baseline values.

### Comparative analysis of thyroid function and thyroglobulin levels in efficacy response and non-efficacy response groups before and after radioactive iodine therapy (RAIT)

**Figure 1:**
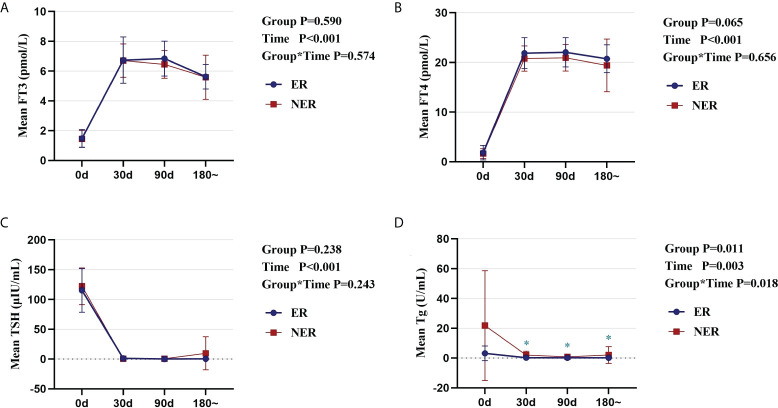
Comparative analysis of the dynamic changes and differences in thyroid function and Tg in ER and NER groups before and after RAIT. Changes in serum FT3 **(A)**, FT4 **(B)**, TSH **(C)**, and Tg **(D)** in the two groups of patients before and after RAIT. ER, excellent response; NER, non-excellent response; RAIT, radioactive iodine treatment; Tg, serum thyroglobulin; d, day; 180~, expressed as 6 months to 1 year after RAIT.

To exclude the possibility of TSH-suppressing treatment influencing the study, we compared thyroid function in the two groups before and after RAIT. Serum FT3 and FT4 levels in the ER and NER groups rose swiftly after treatment with levothyroxine sodium pills, whereas TSH levels quickly decreased, and thereafter, thyroid function stabilized (**Figures 1A–C**). Simultaneously, no significant changes in thyroid function were identified between the two groups (*p* > 0.05; **Figures 1A–C**). The variations in serum Tg levels in the two groups were also studied before and after therapy. The results indicated that Tg levels in all participants fell immediately and stabilized following therapy. From 30 days to 180 days after RAIT, patients in the ER group showed significantly lower Tg levels than those in the NER group (*p* < 0.05; **Figure 1D**). Although there was no significant difference in Tg levels between the two groups before RAIT, serum Tg levels in the NER group tended to be higher than that in the ER group. As a result, lower baseline Tg levels are closely linked to RAIT effectiveness.

### 3.3 Comparative analysis of the distribution of CD4^+^ T cell subsets in pre-RAIT ER, NER, total DTC, and HC groups

**Figure 2:**
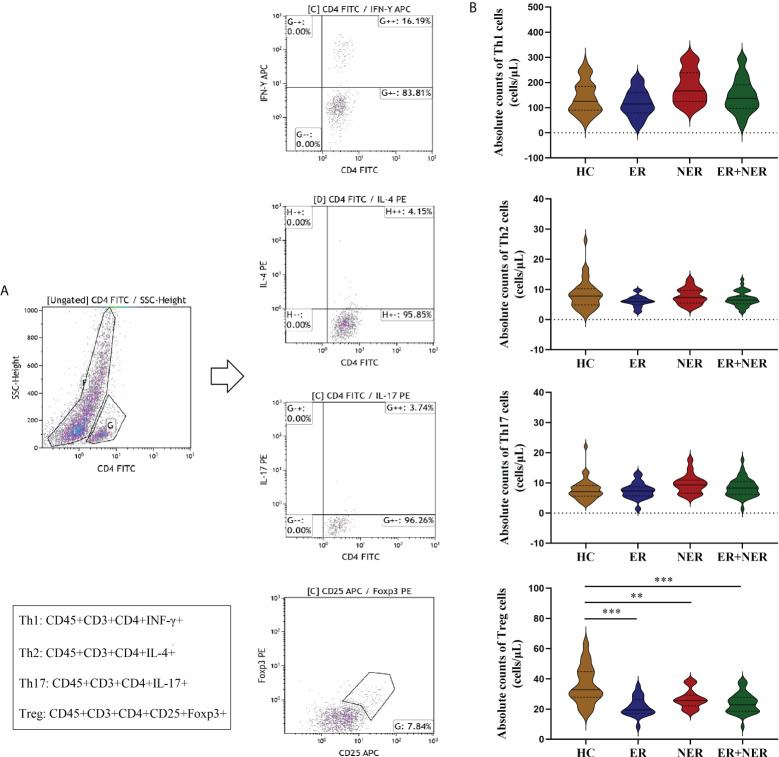
Comparative analysis of circulating CD4^+^ T cell subsets (Th1, Th2, Th17, and Treg cells) between patients with ER, NER, and all DTC patients (ER + NER) before RAIT and HC.**(A)** Flow cytometric analysis of representative CD4^+^ T cell subsets. **(B)** The absolute counts of circulating CD4^+^ T cell subsets in each group of patients before RAIT. ER, excellent response; NER, non-excellent response; RAIT, radioactive iodine treatment; DTC, differentiated thyroid cancer; HC, healthy controls. **p < 0.01, ***p < 0.001.

In all DTC patients (ER + NER), the proportion of Th17 cells (*p* < 0.05; **Figure S2C**) was significantly higher, while the proportion of Treg cells (*p* < 0.001; **Figure S2D**) was significantly lower, compared to the HC group. The proportions of Th1 and Th2 cells were not significantly different between the two groups (*p* > 0.05; **Figures S2A, B**). The proportion of Treg cells was significantly lower in the ER group compared with that of the HC group (*p* = 0.001; **Figure S2D**). The proportion of Th17 cells was significantly higher in the NER group compared to the HC group (*p* = 0.037; **Figure S2C**), whereas that of Treg cells was significantly lower (*p* = 0.013; **Figure S2D**). Furthermore, relative to the HC group, the proportion of Th1 cells in the NER group was significantly higher (*p* = 0.005; **Figure S2A**). The proportional distribution of CD4^+^ T cell subsets in the ER and NER groups was compared, and it was discovered that there was no significant difference between the two groups (*p* > 0.05; **Figure S2**). We then examined how the absolute levels of circulating CD4^+^ T cell subsets differed between the different patient groups. In all DTC patients, we found a substantial decrease only in the absolute numbers of circulating Treg cells relative to those in the HC group (*p* < 0.05; **Figure 2B**). This may be related to tumor burden.

### 3.4 Dynamic observation of the distribution characteristics of CD4^+^ T cell subsets before and after RAIT in ER and NER groups

Compared with pre-RAIT, the proportions of circulating CD4^+^ T cell subsets in ER, NER, and all DTC patients were not significantly altered 30 d after RAIT (*p* > 0.05; **Figures S3A–D**). The proportion of circulating Th1 cells in ER, NER, and all DTC patients was significantly reduced 90 days after RAIT (*p* < 0.05, **Figure S3A**). The proportion of circulating Th2 cells in NER was also significantly reduced 90 days after RAIT (*p* < 0.05; **Figure S3B**). We also compared and analyzed the proportional differences of circulating CD4^+^ T cell subsets between ER and NER patients. Before RAIT, NER patients had a much larger proportion of Th1 cells than ER patients (*p* = 0.041; **Figure S3E**). 30 days after RAIT, the proportion of Th1 cells in the two groups remained significantly different (*p* = 0.019; **Figure S3E**). During the 90 days after RAIT, numbers of circulating Th1 cells of all participants increased steadily over time before dramatically decreasing.

**Figure 3:**
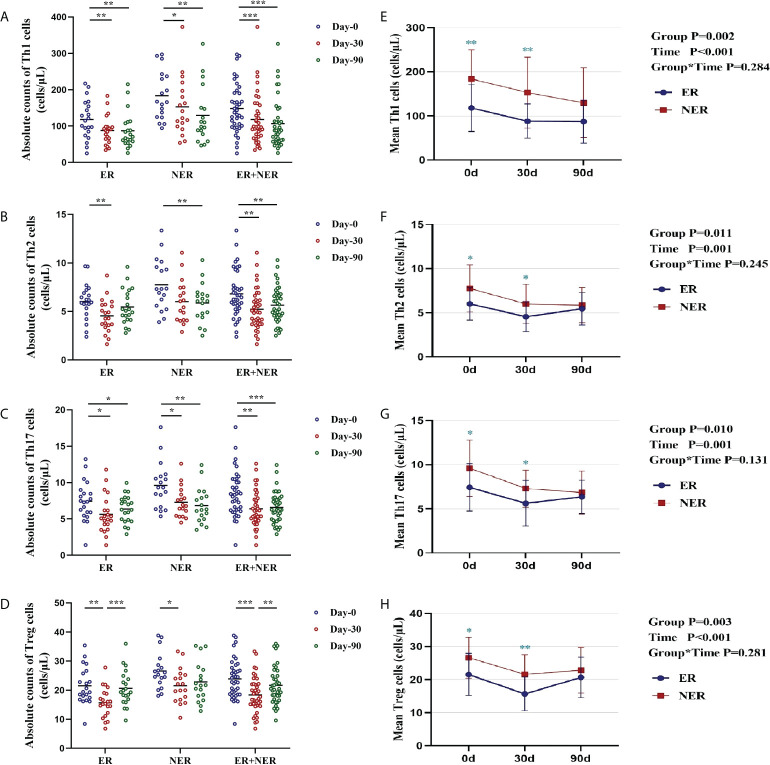
Dynamic changes and differences in absolute counts of circulating CD4^+^ T cell subsets in patients with ER, NER, and all DTC patients (ER+NER) before and after RAIT. **(A–D)** Dynamic changes in the absolute counts of circulating CD4^+^ T cell subsets before and after RAIT in each group. **(E–H)** Differences in the distribution and changes of absolute CD4^+^ T cell subsets counts before and after RAIT in the ER and NER groups. *p < 0.05, **p < 0.01, ***p < 0.001. ER, excellent response; NER, non-excellent response; RAIT, radioactive iodine treatment.

Subsequently, we used dynamic analysis to analyze changes in absolute circulating CD4^+^ T cell subset numbers before and after RAIT. The absolute numbers of all circulating CD4^+^ T cell subsets in both the ER and total DTC groups were significantly lower 30 days after RAIT than those pre-RAIT (*p* < 0.05; **Figures 3A–D**). In the NER group, the absolute numbers of circulating Th1, Th17, and Treg cells were significantly lower 30 days after RAIT than pre-RAIT (*p* < 0.05; **Figures 3A, C, D**). 90 days after RAIT, the absolute numbers of circulating Treg cells in the ER and total DTC groups were significantly greater than those 30 days after RAIT (*p* < 0.05; **Figure 3D**). 90 days after RAIT, the absolute counts of circulating Th1, Th2, and Treg cells in the NER group and total DTC groups were significantly lower than those before RAIT (both *p* < 0.05; **Figures 3A–C**). Surprisingly, we discovered that the absolute Th1 and Th17 cell counts in the ER group were lower 90 days after RAIT than before RAIT (*p* < 0.05; **Figures 3A, C**), but we observed a recovery trend for both cell types. This indicated that in the ER group, circulating CD4^+^ T cells had begun to recover to pre-RAIT levels. This might be a key factor underlying better prognoses in ER patients. In the NER group, however, the majority of circulating CD4^+^ T cell subsets had not recovered, which might be due to persistent tumors and/or metastases during RAIT (**Figures 3A–D**). Next, we investigated the differences in the absolute CD4^+^ T cell counts between the ER and NER groups. Results indicated that numbers of the CD4^+^ T cell subsets in the ER group were considerably lower than those in the NER group before RAIT (*p* < 0.05; **Figures 3E–H**). Additionally, 30 days after RAIT, those of the ER group remained significantly lower than those of the NER group (*p* < 0.05; **Figures 3E–H**). The absolute counts of circulating CD4^+^ T cell subsets did not differ significantly between the two groups 90 days after RAIT (both *p* > 0.05; **Figure 3E–H**). This shows that whereas the baseline numbers of circulating CD4^+^ T cell subsets in the ER group were much lower than those of the NER group, each circulating CD4^+^ T cell subgroup had a greater potential to adapt and recover in the ER group. This might be because patients in the NER group had non- or low-functioning CD4^+^ T cell subsets, and/or CD4^+^ T cell subsets which were resistant to the body’s immune regulatory systems. The molecular pathways underlying these results should be investigated further.

### 3.5 Comparative analysis of the predictive value of CD4^+^ T cell subsets and Tg levels

Univariate analysis revealed that the effectiveness responses (ER and NER) of RAIT were significantly associated with each CD4^+^ T cell subgroup before and 30 days after RAIT in the entire population (*p* < 0.05; **Table 2**). We did not conduct univariate analyses on CD4^+^ T cell subsets 90 days after RAIT since there were no significant differences between the two groups. Our data also revealed that efficacy response was significantly associated with pre-RAIT Tg levels (β = 0.107, *p* = 0.024; **Table 2**), which is consistent with prior findings (14).

**Table 2:** Univariate analysis of prognosis in DTC patients.

Variable	β	Wald	OR	95% CI	P Value
Tg	0.107	5.116	1.113	1.014-1.221	0.024
Th1	0.019	7.264	1.019	1.005-1.033	0.007
Th2	0.362	4.522	1.436	1.029-2.004	0.033
Th17	0.275	4.204	1.316	1.012-1.712	0.04
Treg	0.135	4.92	1.145	1.016-1.291	0.027
Th1-30	0.022	6.735	1.022	1.005-1.039	0.009
Th2-30	0.409	4.309	1.505	1.023-2.214	0.038
Th17-30	0.311	3.999	1.365	1.006-1.851	0.046
Treg-30	0.204	6.979	1.226	1.054-1.427	0.008

Tg, serum thyroglobulin; Th1-30, Th2-30, Th17-30, and Treg-30 represent quantification of CD4^+^ T cell subsets on day 30 after RAIT.

### 3.6 Construction and evaluation of multiple predictive models

Using a multivariate approach, we evaluated the absolute number of immune cells both before and after RAIT as independent risk variables for RAIT success. Then, several prediction models were developed and assessed using Tg together with other risk variables.

#### 3.6.1 Predictive model based on pre-RAIT CD4^+^ T cell subset absolute count and Tg levels

**Figure 4:**
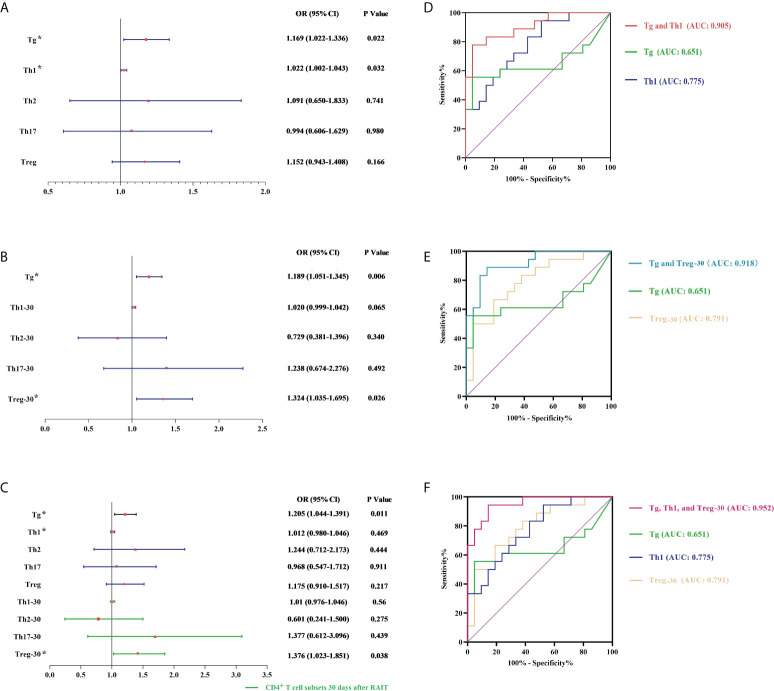
Multivariate logistic analysis and receiver operating characteristic (ROC) analysis. **(A, D)** Model 1 is based on pre-RAIT Tg and absolute counts of pre-RAIT CD4^+^ T cell subsets. **(B, E)** Model 2 is based on pre-RAIT Tg and absolute counts of CD4^+^ T cell subsets 30 d after RAIT. **(C, F)** Model 3 is based on pre-RAIT Tg and absolute counts of CD4^+^ T cell subsets before and 30 d after RAIT. RAIT, radioactive iodine treatment; Tg, serum thyroglobulin. * represents the parameters of the model establishment.

Even after controlling for the interaction between these two factors, Tg levels and absolute pre-RAIT Th1 cell counts were found to be independently associated with RAIT success using logistic multiple regression analysis (**Figure 4A**). The optimum cut-off values separating ER and NER patients, according to ROC curve measurement, were 5.82 g/L (AUC = 0.651, β = 0.099) and 100.73 cells/L (AUC = 0.775, β = 0.073). The ROC curve findings showed that the combined model including pre-RAIT CD4^+^ T cell subset count and Tg levels (Model 1; AUC = 0.905, β = 0.048) had a better predictive capacity than the two indicators alone (**Figure 4B**).

#### 3.6.2 Predictive model based on CD4^+^ T cell subset absolute count 30 days after RAIT and Tg levels

According to logistic multiple regression analysis, the absolute numbers of Treg cells 30 days after RAIT and Tg levels were independently related to RAIT success. The ROC curve revealed that the appropriate cut-off for the absolute Treg cell count 30 days after RAIT was 19.04 cells/L (AUC = 0.791, β = 0.073). Furthermore, the ROC curve findings revealed that the combined model including CD4+ T cell subset absolute count 30 days after RAIT and Tg levels (Model 2; AUC = 0.918, β = 0.043) had a better predictive ability than each index individually (see **Figure 4D**).

#### 3.6.3 Predictive model based on Th1 before RAIT, Treg 30 days after RAIT, and Tg levels

According to a logistic multiple regression analysis, Tg levels and absolute Treg numbers 30 days after RAIT were independently associated with RAIT success (**Figure 4**). Considering the predictive power of Model 1, we made a combined prediction model using pre-RAIT Th1 levels, Treg levels 30 days after RAIT, and Tg levels (Model 3), which had better predictive power (AUC = 0.952, β = 0.03) than the individual predictors and Models 1 and 2 (see **Figure 4F**).

## 4 Discussion

The distribution of circulating CD4^+^ T cells before and after RAIT in DTC patients with various treatment outcomes was dynamically analyzed for the first time, to the best of our knowledge, in this study. We also compared differences in general patient data between the two patient groups.

First, the impact of TSH-suppressing treatment in the two patient groups was investigated. There were no significant differences in the levels of FT3, FT4, or TSH between the two groups. This confirmed that the study’s findings were not impacted by differential TSH levels. In addition, a comparison of Tg levels between the ER and NER groups revealed that lower baseline Tg levels were directly connected to the efficiency of RAIT (14, 35, 36).

We examined the responses of circulating CD4^+^ T cell subsets to RAIT in 39 DTC patients with varying therapeutic outcomes. A prior study discovered that the proportion of circulating Treg cells in DTC patients was considerably higher one month after RAIT compared to before RAIT (37). This, however, contradicts our observations. This might be due to the small sample size of our study as well as the high variability. The absolute numbers of all circulating CD4^+^ T cell subsets were reduced by various degrees in both ER and NER groups 30 days following RAIT, indicating that RAIT may induce some immune suppression; however, this effect is transient and indiscriminate. We discovered that 90 days after RAIT, the circulating CD4^+^ T cell subsets in the ER group had mostly recovered to pre-RAIT levels compared with those in the NER group which did not show similar recovery trends. This shows that circulating CD4^+^ T cells in ER patients are more adaptable and resilient, likely because patients in the NER group had a higher proportion of non- or hypofunctioning CD4^+^ T cell subsets, as well as immune system resistance. Surprisingly, we discovered that 90 days after RAIT, the absolute amount of circulating Th1 cells in the NER group continued to fall. These findings are consistent with the role of Th1 cells in anti-tumor immunity (38–41). Additionally, this study discovered that changes in circulating CD4^+^ T cell subsets in the total DTC group were the result of a combination of trends in both ER and NER patients. This implies that the reported immunological phenomena do not adequately clarify the immune condition of DTC patients if treatment effectiveness is not differentiated. Exploring the distribution patterns of circulating CD4^+^ T cell subsets in DTC patients with various treatment outcomes is thus beneficial for targeted and precise immunotherapy.

Simultaneously, we looked at the differences in circulating CD4^+^ T cell subset distribution between the two groups of patients before and after RAIT. Before and 30 days after RAIT, our findings revealed substantial disparities in circulating immune cells, and patients with lower absolute numbers of CD4^+^ T cell subsets had a better prognosis, consistent with previous studies (42). Patient prognosis is much poorer when CD4^+^ T cells are heavily proliferated, which might imply the existence of highly inflammatory residuals or recurring foci of highly inflammatory malignancies which lack specific immunogenic targets.

The importance of investigating absolute immune cell numbers is emphasized in this work. As opposed to proportions, absolute quantitative research can more accurately and comprehensively depict the state of immunological alterations. This has been demonstrated in several immunological disorders (43–45).

Finally, we developed and tested three different predictive models. When compared with the individual metrics, the performance of the three predictive models were superior. Model 3 had a greater AUC than the first two, indicating greater predictive power. However, we recommend the first two models due to their economic benefits. Model 1 is easier to manage, but Model 2 gives predictive data as well as the immunological state of patients following RAIT. Therefore, clinicians can select the best-suited model for their requirements.

This study has some limitations: 1) the sample size is small; 2) the present study does not rule out the effects of hypothyroidism before RAIT on the immunological function of DTC patients; 3) the study did not perform further study of tumor tissues and tumor microenvironments. Therefore, prospective, large-sample, multi-center investigations are needed in the future, and the impact of pre-RAIT hypothyroidism on DTC patient immunological function should not be ruled out.

In summary, our data demonstrate, for the first time, that circulating immune cell profiles in DTC patients are highly heterogeneous. These differences can be exploited to predict patient prognosis and develop more targeted treatment regimens. Fewer absolute counts of circulating immune cells are linked to improved clinical outcomes as well as stronger adaptive and resilience capacities. Moreover, differences in the tumor microenvironment appear to play an integral role (46–50). As a result, more research is needed to differentiate individuals with high absolute T cell counts from those with low absolute T cell counts. In the future, single-cell sequencing and gene expression profiling approaches should be coupled to properly characterize the distinctions between the two. Furthermore, in DTC patients, RAIT may produce transient and indiscriminate radiation damage to circulating immune cells, which may have implications that we do not currently understand. Finally, we built various predictive models using the high predictive power of CD4^+^ T cell subsets to offer more accurate prognostic information.

## Data availability statement

The raw data supporting the conclusions of this article will be made available by the authors, without undue reservation.

## Ethics statement

The studies involving human participants were reviewed and approved by The institutional review board of Shanxi Medical University’s First Hospital in China approved this study. The patients/participants provided their written informed consent to participate in this study.

## Author contributions

All authors contributed to data acquisition. Z-YS and S-XZ provided the study design. C-HL, Z-HC, DF, YX, L-XW, and K-YL performed the sample collection and completed the patient communication. Z-FW contributed to the discussion. Z-YS analyzed the data and wrote the manuscript. X-FL, H-YL, and S-JL critically revised the manuscript. All authors were responsible for data analysis and manuscript preparation and approve its submission to this journal.

## Funding

This work was supported by grants from the National Natural Science Foundation of China (No. 82001740), the Ministry of Education of the People’s Republic of China, Personnel and Social Affairs Department of Shanxi Province (No. 202003), Department of Finance of Shanxi Province (No. 2019023), and the Science and Technology Department of Shanxi Province (No. 201804D131042).

## Acknowledgments

We sincerely appreciate the generous help from the Collaborative Innovation Center for Molecular Imaging of Precision Medicine.

## Conflict of interest

The authors declare that the research was conducted in the absence of any commercial or financial relationships that could be construed as potential conflict of interest.

## Publisher’s note

All claims expressed in this article are solely those of the authors and do not necessarily represent those of their affiliated organizations, or those of the publisher, the editors and the reviewers. Any product that may be evaluated in this article, or claim that may be made by its manufacturer, is not guaranteed or endorsed by the publisher.

## References

[1] Bray F Ferlay J Soerjomataram I Siegel R Torre L Jemal A. Global cancer statistics 2018: GLOBOCAN estimates of incidence and mortality worldwide for 36 cancers in 185 countries. CA: Cancer J Clin (2018) 68:394–424. doi: 10.3322/caac.21492 30207593

[2] Avram AM Zukotynski K Nadel HR Giovanella L. Management of differentiated thyroid cancer: The standard of care. J Nucl Med (2022) 63:189–95. doi: 10.2967/jnumed.121.262402 34413146

[3] Laaksonen M MacInnis R Canfell K Shaw J Magliano D Banks E. Thyroid cancers potentially preventable by reducing overweight and obesity in Australia: A pooled cohort study. Int J Cancer (2022) 150:1281–90. doi: 10.1002/ijc.33889 34847246

[4] Filetti S Durante C Hartl D Leboulleux S Locati L Newbold K. Thyroid cancer: ESMO clinical practice guidelines for diagnosis, treatment and follow-up†. Ann Oncol Off J Eur Soc Med Oncol (2019) 30:1856–83. doi: 10.1093/annonc/mdz400 31549998

[5] Tuttle R Ahuja S Avram A Bernet V Bourguet P Daniels G. Controversies, consensus, and collaboration in the use of I therapy in differentiated thyroid cancer: A joint statement from the American thyroid association, the European association of nuclear medicine, the society of nuclear medicine and molecular imaging, and the European thyroid association. Thyroid Off J Am Thyroid Assoc (2019) 29:461–70. doi: 10.1089/thy.2018.0597 30900516

[6] Haugen B Alexander E Bible K Doherty G Mandel S Nikiforov Y. 2015 American Thyroid association management guidelines for adult patients with thyroid nodules and differentiated thyroid cancer: The American thyroid association guidelines task force on thyroid nodules and differentiated thyroid cancer. Thyroid Off J Am Thyroid Assoc (2016) 26:1–133. doi: 10.1089/thy.2015.0020 PMC4739132 26462967

[7] Toubeau M Touzery C Arveux P Chaplain G Vaillant G Berriolo A. Predictive value for disease progression of serum thyroglobulin levels measured in the postoperative period and after (131)I ablation therapy in patients with differentiated thyroid cancer. J Nucl Med Off publication Soc Nucl Med (2004) 45:988–94. 15181134

[8] Cheng L Sa R Luo Q Fu H Jin Y Tang L. Unexplained hyperthyroglobulinemia in differentiated thyroid cancer patients as an indication for radioiodine adjuvant therapy: A prospective multicenter study. J Nucl Med Off publication Soc Nucl Med (2021) 62:62–8. doi: 10.2967/jnumed.120.243642 32358095

[9] Piccardo A Arecco F Puntoni M Foppiani L Cabria M Corvisieri S. Focus on high-risk DTC patients: high postoperative serum thyroglobulin level is a strong predictor of disease persistence and is associated to progression-free survival and overall survival. Clin Nucl Med (2013) 38:18–24. doi: 10.1097/RLU.0b013e318266d4d8 23242039

[10] Durante C Haddy N Baudin E Leboulleux S Hartl D Travagli J. Long-term outcome of 444 patients with distant metastases from papillary and follicular thyroid carcinoma: benefits and limits of radioiodine therapy. J Clin Endocrinol Metab (2006) 91:2892–9. doi: 10.1210/jc.2005-2838 16684830

[11] Capdevila J Awada A Führer-Sakel D Leboulleux S Pauwels P. Molecular diagnosis and targeted treatment of advanced follicular cell-derived thyroid cancer in the precision medicine era. Cancer Treat Rev (2022) 106:102380. doi: 10.1016/j.ctrv.2022.102380 35305441

[12] Raue F Frank-Raue K. Thyroid cancer: Risk-stratified management and individualized therapy. Clin Cancer Res an Off J Am Assoc Cancer Res (2016) 22:5012–21. doi: 10.1158/1078-0432.CCR-16-0484 27742787

[13] Schlumberger M Leboulleux S. Current practice in patients with differentiated thyroid cancer. Nat Rev Endocrinol (2021) 17:176–88. doi: 10.1038/s41574-020-00448-z 33339988

[14] Campennì A Ruggeri R Siracusa M Comis A Romano D Vento A. Early preablation rhTSH-stimulated thyroglobulin predicts outcome of differentiated thyroid cancer (DTC) patients. Eur J Nucl Med Mol Imaging (2021) 48:2466–75. doi: 10.1007/s00259-020-05153-7 33416957

[15] Song E Oh H Jeon M Chung K Hong S Ryu J. The value of preoperative antithyroidperoxidase antibody as a novel predictor of recurrence in papillary thyroid carcinoma. Int J Cancer (2019) 144:1414–20. doi: 10.1002/ijc.31944 30357831

[16] Nascimento C Ferreira F. Tumor microenvironment of human breast cancer, and feline mammary carcinoma as a potential study model. Biochim Biophys Acta Rev Cancer (2021) 1876:188587. doi: 10.1016/j.bbcan.2021.188587 34237352

[17] Desai R Coxon A Dunn G. Therapeutic applications of the cancer immunoediting hypothesis. Semin Cancer Biol (2022) 78:63–77. doi: 10.1016/j.semcancer.2021.03.002 33711414

[18] Baxevanis C Fortis S Perez S. The balance between breast cancer and the immune system: Challenges for prognosis and clinical benefit from immunotherapies. Semin Cancer Biol (2021) 72:76–89. doi: 10.1016/j.semcancer.2019.12.018 31881337

[19] O'Donnell J Teng M Smyth M. Cancer immunoediting and resistance to T cell-based immunotherapy. Nat Rev Clin Oncol (2019) 16:151–67. doi: 10.1038/s41571-018-0142-8 30523282

[20] Schietinger A Philip M Liu R Schreiber K Schreiber H. Bystander killing of cancer requires the cooperation of CD4(+) and CD8(+) T cells during the effector phase. J Exp Med (2010) 207:2469–77. doi: 10.1084/jem.20092450 PMC2964573 20921286

[21] Binnewies M Mujal A Pollack J Combes A Hardison E Barry K. Unleashing type-2 dendritic cells to drive protective antitumor CD4 T cell immunity. Cell (2019) 177:556–71.e16. doi: 10.1016/j.cell.2019.02.005 30955881 PMC6954108

[22] Borst J Ahrends T Bąbała N Melief C Kastenmüller W. CD4 T cell help in cancer immunology and immunotherapy. Nat Rev Immunol (2018) 18:635–47. doi: 10.1038/s41577-018-0044-0 30057419

[23] Quezada S Simpson T Peggs K Merghoub T Vider J Fan X. Tumor-reactive CD4(+) T cells develop cytotoxic activity and eradicate large established melanoma after transfer into lymphopenic hosts. J Exp Med (2010) 207:637–50. doi: 10.1084/jem.20091918 PMC2839156 20156971

[24] Alspach E Lussier DM Miceli AP Kizhvatov I DuPage M Luoma AM. MHC-II neoantigens shape tumour immunity and response to immunotherapy. Nature (2019) 574:696–701. doi: 10.1038/s41586-019-1671-8 31645760 PMC6858572

[25] Binnewies M Mujal AM Pollack JL Combes AJ Hardison EA Barry KC. Unleashing type-2 dendritic cells to drive protective antitumor CD4(+) T cell immunity. Cell (2019) 177:556–71.e16. doi: 10.1016/j.cell.2019.02.005 30955881 PMC6954108

[26] Ding ZC Liu C Cao Y Habtetsion T Kuczma M Pi W. IL-7 signaling imparts polyfunctionality and stemness potential to CD4(+) T cells. Oncoimmunology (2016) 5:e1171445. doi: 10.1080/2162402X.2016.1171445 27471650 PMC4938319

[27] Huang A Postow M Orlowski R Mick R Bengsch B Manne S. T-Cell invigoration to tumour burden ratio associated with anti-PD-1 response. Nature (2017) 545:60–5. doi: 10.1038/nature22079 PMC5554367 28397821

[28] Larkin J Chiarion-Sileni V Gonzalez R Grob J Cowey C Lao C. Combined nivolumab and ipilimumab or monotherapy in untreated melanoma. New Engl J Med (2015) 373:23–34. doi: 10.1056/NEJMoa1504030 26027431 PMC5698905

[29] Topalian S Sznol M McDermott D Kluger H Carvajal R Sharfman W. Survival, durable tumor remission, and long-term safety in patients with advanced melanoma receiving nivolumab. J Clin Oncol Off J Am Soc Clin Oncol (2014) 32:1020–30. doi: 10.1200/JCO.2013.53.0105 PMC4811023 24590637

[30] Miggelbrink A Jackson J Lorrey S Srinivasan E Waibl-Polania J Wilkinson D. CD4 T-cell exhaustion: Does it exist and what are its roles in cancer? Clin Cancer Res an Off J Am Assoc Cancer Res (2021) 27:5742–52. doi: 10.1158/1078-0432.CCR-21-0206 PMC8563372 34127507

[31] Kothalawala W Győrffy B. Transcriptomic and cellular content analysis of colorectal cancer by combining multiple independent cohorts. Clin Trans Gastroenterol (2022). doi: 10.14309/ctg.0000000000000517 PMC9945259 35858620

[32] Revilla G Cedó L Tondo M Moral A Pérez J Corcoy R. LDL, HDL and endocrine-related cancer: From pathogenic mechanisms to therapies. Semin Cancer Biol (2021) 73:134–57. doi: 10.1016/j.semcancer.2020.11.012 33249202

[33] Revilla G Pons M Baila-Rueda L García-León A Santos D Cenarro A. Cholesterol and 27-hydroxycholesterol promote thyroid carcinoma aggressiveness. Sci Rep (2019) 9:10260. doi: 10.1038/s41598-019-46727-2 31311983 PMC6635382

[34] Targher G Montagnana M Salvagno G Moghetti P Zoppini G Muggeo M. Association between serum TSH, free T4 and serum liver enzyme activities in a large cohort of unselected outpatients. Clin Endocrinol (2008) 68:481–4. doi: 10.1111/j.1365-2265.2007.03068.x 17941901

[35] Jeong E Yoon J Lee S Soh E Lee J An Y. Risk factors for indeterminate response after radioactive iodine therapy in patients with differentiated thyroid cancer. Clin Nucl Med (2019) 44:714–8. doi: 10.1097/RLU.0000000000002653 31162260

[36] Nóbrega G Cavalcanti M Leite V Vilar L Brandão S. Value of stimulated pre-ablation thyroglobulin as a prognostic marker in patients with differentiated thyroid carcinoma treated with radioiodine. Endocrine (2022) 76:642–7. doi: 10.1007/s12020-022-03021-y 35237910

[37] Jiang L Zhan Y Gu Y Ye Y Cheng Y Shi H. Changes of regulatory T and b cells in patients with papillary thyroid carcinoma after 131I radioablation: a preliminary study. BioMed Res Int (2013) 2013:683768. doi: 10.1155/2013/683768 24350284 PMC3856126

[38] Katsumoto Y Monden T Takeda T Haba A Ito Y Wakasugi E. Analysis of cytotoxic activity of the CD4+ T lymphocytes generated by local immunotherapy. Br J Cancer (1996) 73:110–6. doi: 10.1038/bjc.1996.20 PMC2074300 8554971

[39] Nagore E Virós A Kumar R. Positive attributes of anti-TERT CD4 T-helper type 1 immune responses in melanoma. J Invest Dermatol (2022) 142:279–81. doi: 10.1016/j.jid.2021.09.005 34666894

[40] Herrera F Ronet C Ochoa de Olza M Barras D Crespo I Andreatta M. Low-dose radiotherapy reverses tumor immune desertification and resistance to immunotherapy. Cancer Discovery (2022) 12:108–33. doi: 10.1158/2159-8290.CD-21-0003 PMC9401506 34479871

[41] Noël G Fontsa M Garaud S De Silva P de Wind A Van den Eynden G. Functional Th1-oriented T follicular helper cells that infiltrate human breast cancer promote effective adaptive immunity. J Clin Invest (2021) 131(19):e139905. doi: 10.1172/JCI139905 34411002 PMC8483751

[42] Cheng YK Chen DW Chen P He X Li PS Lin ZS. Association of peripheral blood biomarkers with response to anti-PD-1 immunotherapy for patients with deficient mismatch repair metastatic colorectal cancer: A multicenter cohort study. Front Immunol (2022) 13:809971. doi: 10.3389/fimmu.2022.809971 35185898 PMC8850282

[43] Liu H Shi Z Fan D Zhang S Wu L Lu K. Absolute reduction in peripheral regulatory T cells in patients with graves' disease and post-treatment recovery. Mol Immunol (2022) 144:49–57. doi: 10.1016/j.molimm.2022.02.004 35189399

[44] Zhang S Wang J Sun H Zhang J Liu G Luo J. Circulating regulatory T cells were absolutely decreased in dermatomyositis/polymyositis patients and restored by low-dose IL-2. Ann rheumatic Dis (2019) 80(8):e130. doi: 10.1136/annrheumdis-2019-216246 31611221

[45] An H Li X Li F Gao C Li X Luo J. The absolute counts of peripheral T lymphocyte subsets in patient with ankylosing spondylitis and the effect of low-dose interleukin-2. Medicine (2019) 98:e15094. doi: 10.1097/MD.0000000000015094 30985663 PMC6485794

[46] Wolf Y Samuels Y. Intratumor heterogeneity and anti-tumor immunity shape one another bidirectionally. Clin Cancer Res an Off J Am Assoc Cancer Res (2022) 28(14):2994–3001. doi: 10.1158/1078-0432.CCR-21-1355 PMC9306293 35380639

[47] Zheng Y Wang X Huang M. Metabolic regulation of CD8 T cells: from mechanism to therapy. Antioxidants Redox Signaling (2022). doi: 10.1089/ars.2022.0040 35345890

[48] Wang X Wang H Liu D Wang N He D Wu Z. Deep learning using bulk RNA-seq data expands cell landscape identification in tumor microenvironment. Oncoimmunology (2022) 11:2043662. doi: 10.1080/2162402X.2022.2043662 35251771 PMC8890395

[49] Van den Bossche V Zaryouh H Vara-Messler M Vignau J Machiels J Wouters A. Microenvironment-driven intratumoral heterogeneity in head and neck cancers: clinical challenges and opportunities for precision medicine. Drug resistance updates Rev commentaries antimicrobial Anticancer chemotherapy (2022) 60:100806. doi: 10.1016/j.drup.2022.100806 35121337

[50] Peng W Zhou X Yan W Li Y Du C Wang X. Dissecting the heterogeneity of the microenvironment in primary and recurrent nasopharyngeal carcinomas using single-cell RNA sequencing. Oncoimmunology (2022) 11:2026583. doi: 10.1080/2162402X.2022.2026583 35096485 PMC8794254

