# Supplementary material for: Differential distribution and prognostic value of CD4+ T cell subsets before and after radioactive iodine therapy in differentiated thyroid cancer with varied curative outcomes
Source: Front Immunol. 2022 Aug 26;13:966550. doi: 10.3389/fimmu.2022.966550 (PMC9459039; doi:10.3389/fimmu.2022.966550)
Supplement: Supplementary file 1 [file DataSheet_1.docx]

Supplementary Material

# Supplementary Figures

**
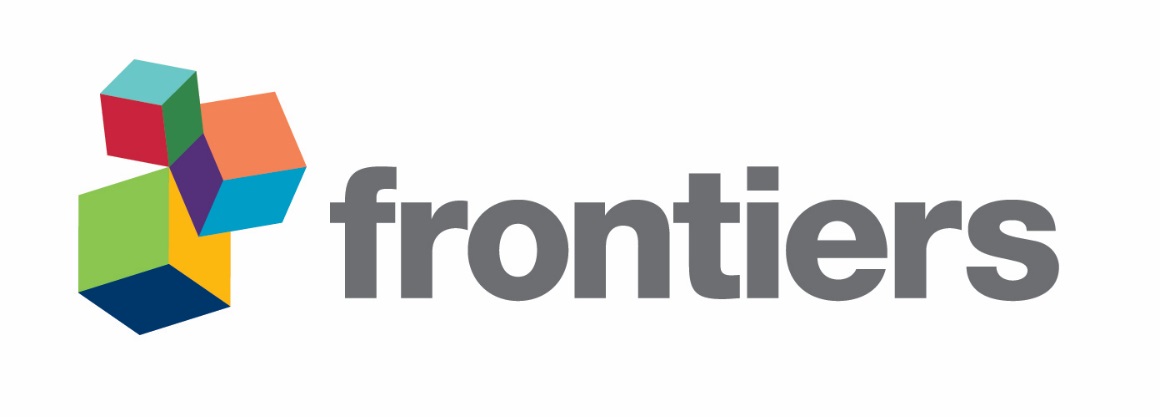
**

## Supplementary Figure S1


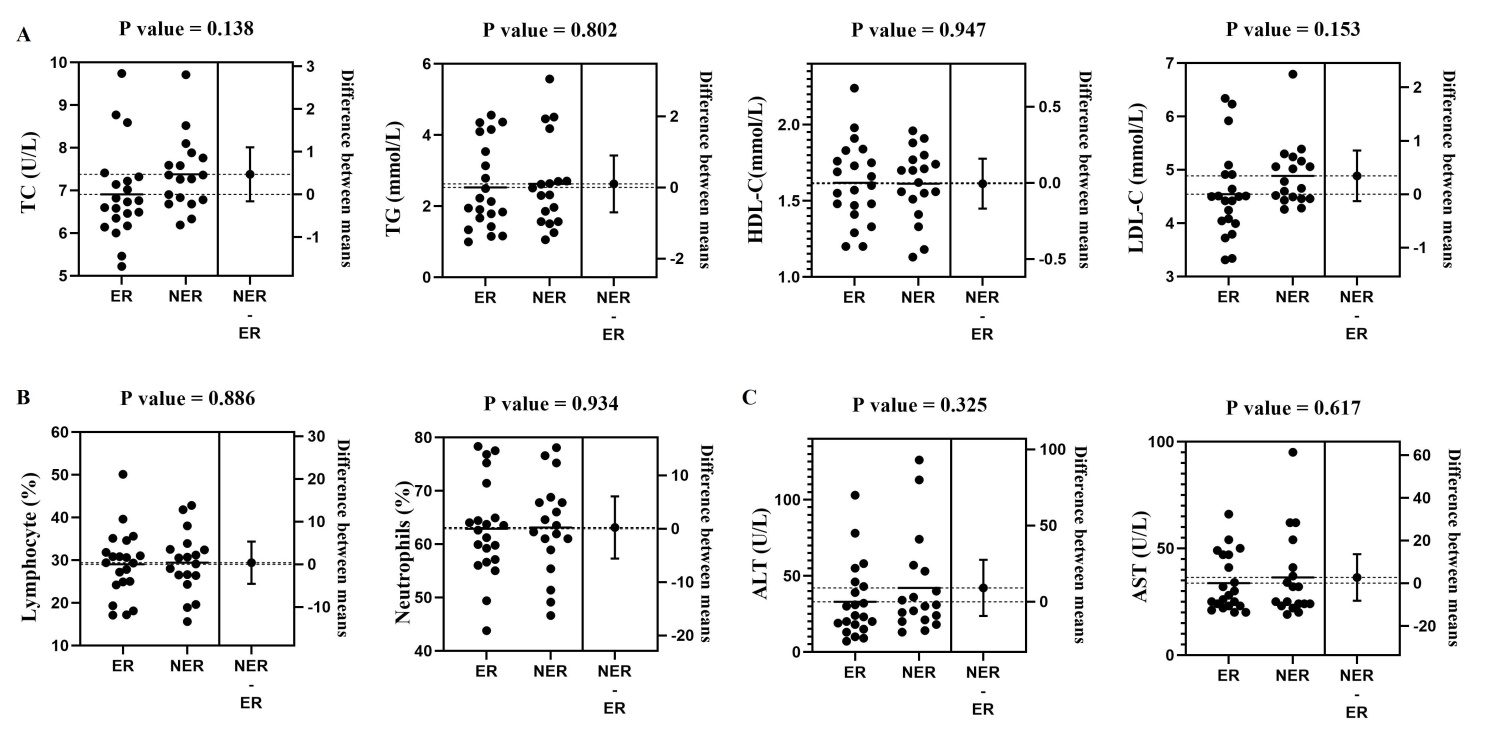


Figure S1. Routine assays for ER and NER groups. The blood lipids (A), liver function (B), lymphocytes, and neutrophils (C) were compared between the two groups before RAIT. *p < 0.05. ER, excellent response; NER, non-excellent response; RAIT, radioactive iodine treatment.

## Supplementary Figure S2

**
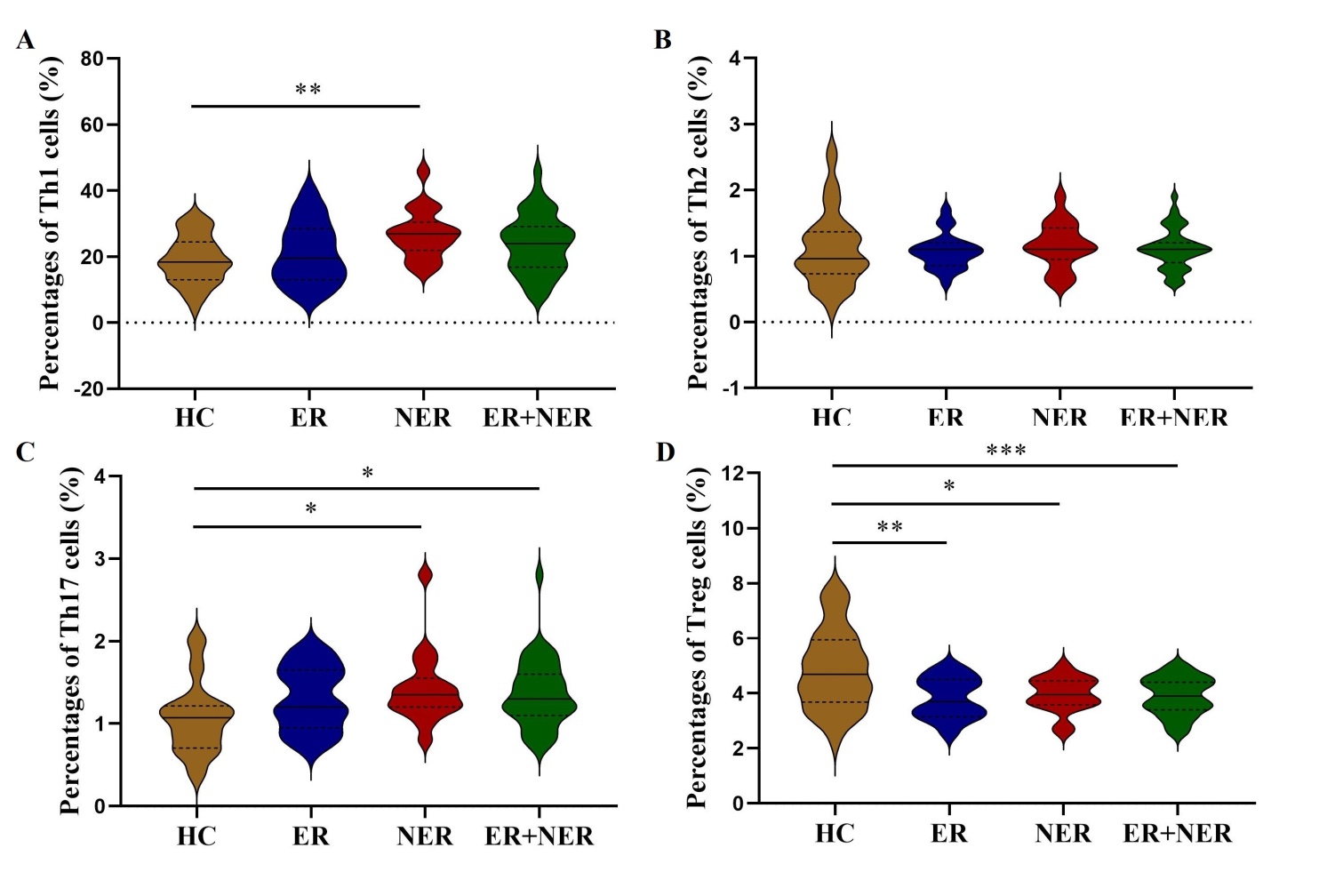
**

Figure S2. Comparative analysis of circulating CD4^+^ T cell subsets (Th1, Th2, Th17, and Treg cells) between patients with ER, NER, and all DTC patients (ER + NER) before RAIT and HC.(A-D) The percentage of circulating CD4^+^ T cell subsets in each group of patients before RAIT. ER, excellent response; NER, non-excellent response; RAIT, radioactive iodine treatment; DTC, differentiated thyroid cancer; HC, healthy controls.

## Supplementary Figure S3


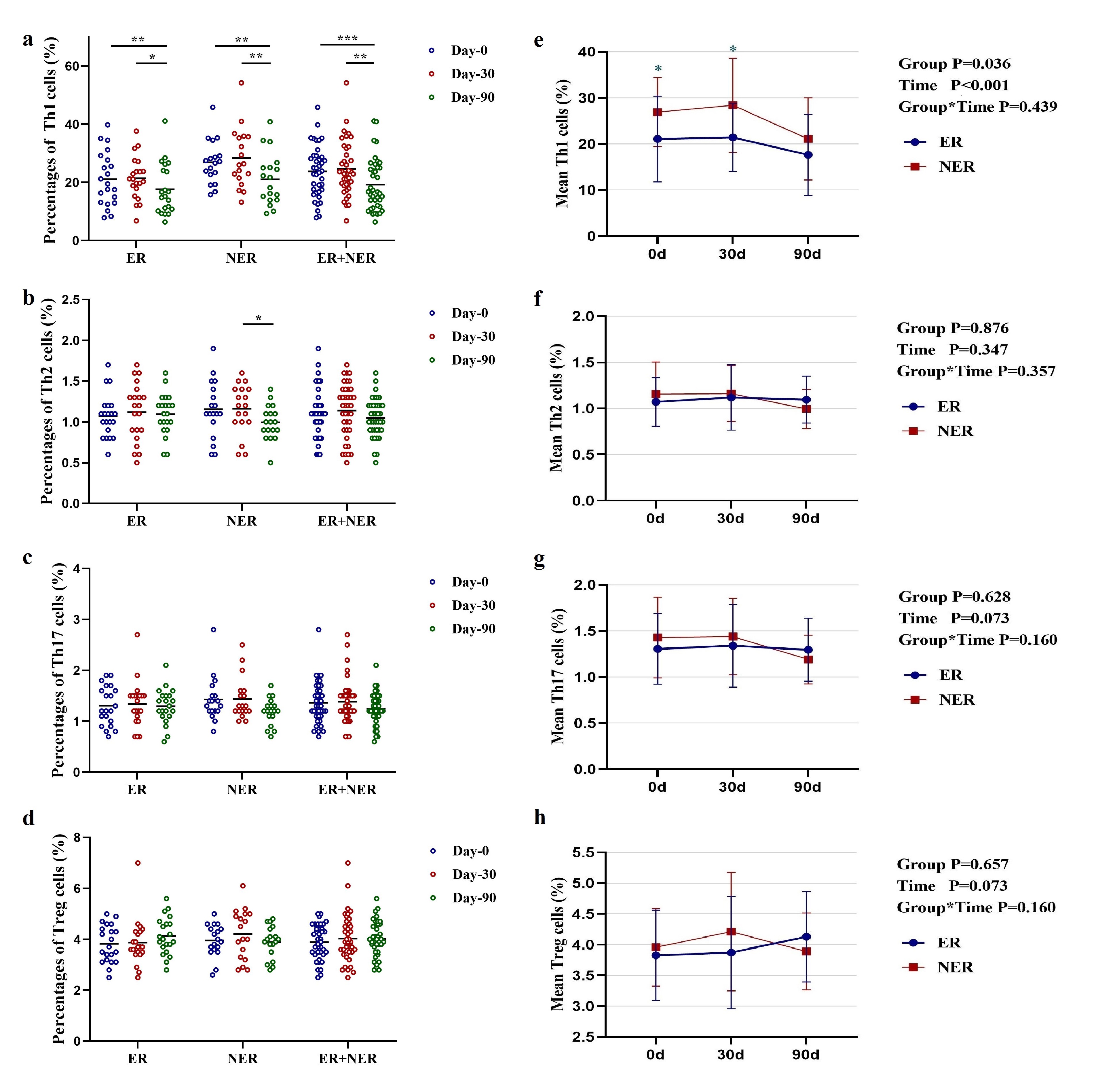


Figure S2. Dynamic changes and differences in the percentage of circulating CD4^+^ T cell subsets in patients with ER, NER, and all DTC patients (ER+NER) before and after RAIT. (**A-D**) Dynamic changes in the percentage of CD4^+^ T cell subsets before and after RAIT in each group. (**E-H**) Differences in the distribution and changes of CD4^+^ T cell subsets before and after RAIT in the ER and NER groups. **p < 0.05, **p* < 0.01, ****p* < 0.001. ER, excellent response; NER, non-excellent response; RAIT, radioactive iodine treatment; DTC, differentiated thyroid cancer.
